# Abdominal massage alleviates functional diarrhea in immature rats via modulation of intestinal microbiota and tight junction protein

**DOI:** 10.3389/fped.2022.922799

**Published:** 2022-07-22

**Authors:** Yanyi Huang, Qing Ma, Jingxin He, Xingshan Liang, Qingxin Mai, Huifang Luo, Jingyi Hu, Yang Song

**Affiliations:** School of Nursing, Guangzhou University of Chinese Medicine, Guangzhou, China

**Keywords:** abdominal massage, functional diarrhea, intestinal microbiota, tight junction protein, immature rat, 16S rDNA amplicon sequencing

## Abstract

Functional diarrhea (FD) is a common type of chronic diarrhea in children. Recurrent diarrhea can negatively impact children's quality of life and raise healthcare costs significantly. However, conventional treatments are ineffective and limited. Moreover, children with chronic conditions have poor medication compliance. Therefore, non-pharmacological and complementary treatments are urgently needed. In China, abdominal massage is widely used to treat diarrhea in children. Numerous clinical studies have verified its usefulness in treating gastrointestinal disorders as well. Nevertheless, its intrinsic mechanisms are still unclear, and the impact of massage direction on treatment effects has received less attention. In our study, we found that FD was not associated with pathogen infection. A dysbiosis of the intestinal microbiota and disruption of the intestinal barrier are most likely to cause FD. Moreover, this study also substantiates that abdominal massage can mitigate functional diarrhea by altering the intestinal microbiota structure and decreasing the number of bacteria that damage intestinal mucosal barriers. The reduction of *Ruminococcus_torques_group* and *Clostridium_innocuum_group* at the genus level potentially mediated the beneficial effects of abdominal massage on alleviating diarrhea. Furthermore, massaging from two different directions, clockwise (CW) and counter-clockwise (CCW) massage, would not significantly influence the effect of the massage on intestinal microbiota or tight junction proteins. In summary, abdominal massage is an effective complementary therapy for children suffering from functional diarrhea.

## Introduction

For children, diarrhea is a prevalent and serious clinical condition (1). Functional diarrhea (FD), the most common type of persistent or chronic diarrhea, is a functional gastrointestinal disorder (2). The Rome IV definition of FD is the presence of loose (mushy) or watery stools four times a day without pain (3). The prevalence of FD is estimated at around 6.4% in the USA and 1.54% in China (4–6). Children who experience recurrent episodes of diarrhea are at greater risk of developing other health conditions, such as under nutrition, diarrheal infections, and immunodeficiency (7). Consequently, those conditions will have a substantial impact on children's quality of life as well as burden the healthcare system.

However, the specific pathogenesis and mechanism of FD remain unclear. In previous studies, intestinal microbiota dysbiosis and intestinal barrier disruption have been suggested as contributing factors to FD (8, 9). In a healthy human intestine, commensal bacteria coexist with pathogenic bacteria in a dynamic balance. It has been suggested that intestinal microbiota dysbiosis (increased numbers of pathogenic bacteria and decreased numbers of commensal bacteria) might result in the disruption of intestinal barriers (10, 11). Tight junction proteins play a vital role in maintaining the integrity of the intestinal barriers by averting the transport of pathogenic bacteria and toxins. Reduced intestinal tight junction proteins can disrupt intestinal barriers, alter intestinal epithelial permeability, eventually cause diarrhea. Recurrent episodes of diarrhea can disrupt the normal intestinal barriers and substantially impair the development of a healthy intestinal microbiota (12). Thus, intestinal microbiota dysbiosis could simultaneously be the cause and consequence of FD.

There are limited conventional treatments available to mitigate the symptoms of FD, such as fluid replacement, antibiotics, and probiotics (13). Children with chronic conditions also have a poor medication compliance rate. Therefore, non-pharmacological and complementary treatments are urgently needed. The abdominal massage, which is a regular and circular movement around the belly button, is widely used for children with diarrhea in China as a simple, safe, and cost-effective method, practiced both in clinics and at home (14, 15). Furthermore, children readily accept abdominal massage as opposed to other types of treatment, such as medication. Many clinical studies have also confirmed that abdominal massage is beneficial for treating common gastrointestinal disorders, such as diarrhea (16), constipation (17), and gastrointestinal dysfunction (18). Consequently, abdominal massage could be an effective complementary therapy for children suffering from FD.

However, preceding evidences regarding its efficacy has been insufficient, and its underlying mechanism has yet to be elucidated. Moreover, we have found that in clinical settings, both clockwise and counter-clockwise massage directions were generally used when a parent massaged a child's abdomen. The direction of massage depends on individual habits, the left-handed people usually use the counter-clockwise direction and the right-handed people generally use the clockwise direction. Nonetheless, researches have been focused less on the impact of massage direction than on treatment outcomes. By analyzing 16S sequencing, we identified substantial changes in intestinal microbiota in rats with FD. After that, we investigated the effects of different directions of abdominal massage on the modulation of intestinal microbiota and intestinal tight junctions. These results may provide useful insight into the therapeutic benefits of abdominal massage for FD.

## Materials and methods

### Animal model

Guangzhou University of Chinese Medicine's Animal Experimentation Ethics Committee approved the experiments (protocol #20210317004). The Animal Experiment Center [SCXK (YUE) 2019-0047] at Guangzhou University of Chinese Medicine supplied 50 specific-pathogen-free Sprague-Dawley rats (age: 3 weeks, weight: 50.30 ± 12.33 g, sex: ratio male: female ratio of 1:1). All animals were housed in standard cages at the Animal Experiment Center of Guangzhou University of Chinese Medicine [SYXK (YUE) 2018-0085]. Growing conditions were as follows: 22 ± 2°C, 40–60% humidity, 12 h light/12 h dark cycle, with food and water provided. The body weights of the animals were observed daily throughout the study.

After 1 week of adaptive feeding, fifty SD rats were randomly divided into five groups, including the control group (*n* = 10), model group (*n* = 10), sham-massage group (*n* = 10), CW-massage group (*n* = 10), and CCW-massage group (*n* = 10). The rats in the control group were fed with a regular standard diet and drank water freely every day. For the rest of the rats, miscible liquids (3 g/ml) of Folium Sennae and Rhubarb were administered intragastrically of 10 ml/kg for 1 week (19, 20). Both Folium Sennae and Rhubarb are Chinese herbal medicines. They were commonly used as laxatives to induce diarrhea models (21). During experiment procedure, six SD rats with successful modeling in each group were finally selected for further investigation (*n* = 6 in each group).

### Abdominal massage

**Figure 1:**
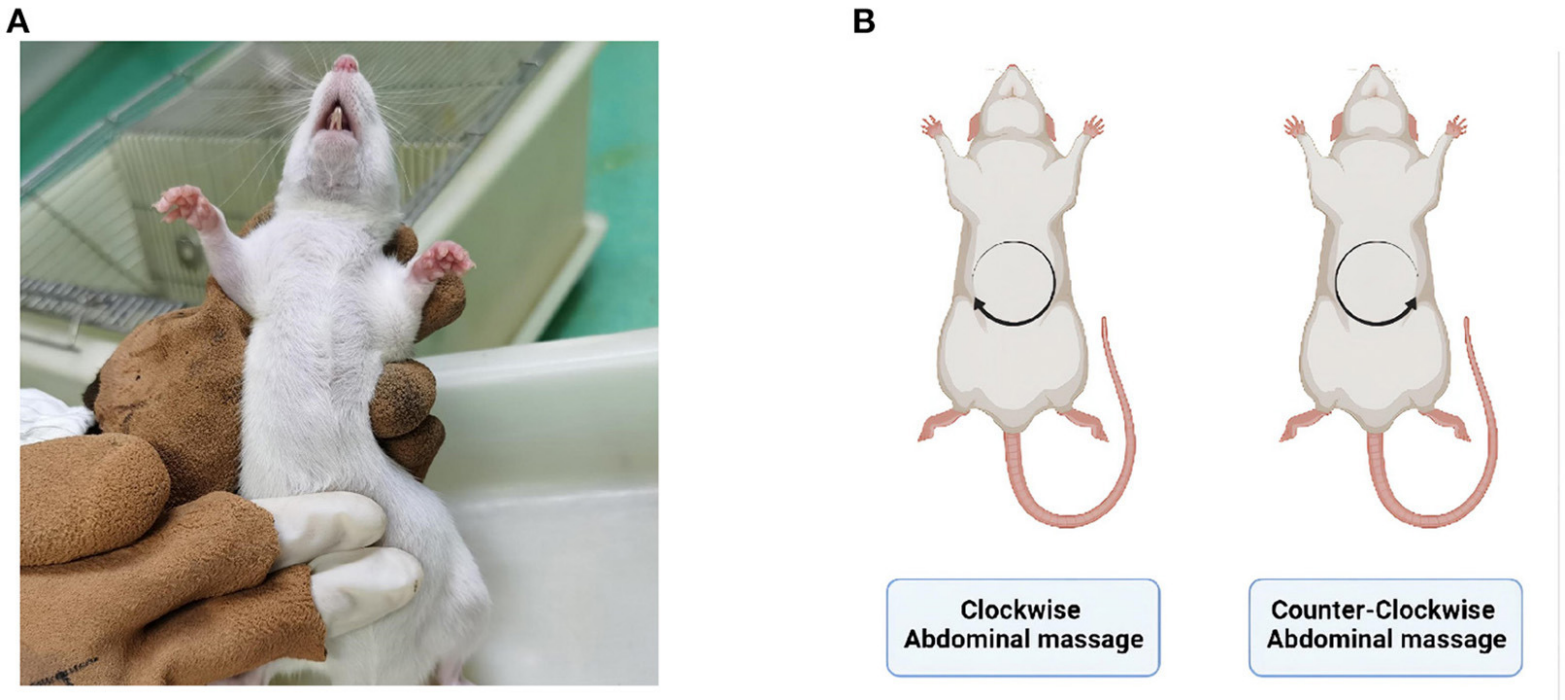
**(A)** Abdominal massage site. **(B)** Schematic of massage direction. Created with BioRender.com.

No treatment was administered to the control and model groups after modeling. The CW-massage group was treated with clockwise abdominal massage, while the CCW-massage group received counter-clockwise abdominal massage (Figures 1A,B). The frequency of the abdominal massage is 120–160 times per minute, with a duration of 10 min once a day for 14 days. In the sham-massage group, irregular touch stimulation was applied with the same frequency. Several points should be considered when massaging: (a) The abdominal massage is applied to the rat's abdomen using the whorl surface of the index and middle fingers, rubbing gently in a circular motion. (b) Should the rats avoid being massaged on their abdomens, the duration of the massage will be extended for 1~2 min to make up for the time loss owing to the rats' avoidance behavior. (c) If the rats struggled violently, the massage would pause and resume when they become calm again. (d) The same researcher would performe all the massage throughout the experiment.

### Sample collection and preparation

On the last day, after 12 h of starvation, the rats were sacrificed by anesthetic overdose intraperitoneally. Four rats (sex: ratio male: female ratio of 1:1) from each group were randomly selected. Samples of colon content were collected and stored in a freezer storage tube, then instantly kept at −80°C until examined for intestine microbiota. We also collected, processed, and froze colon tissues at −80°C for RT-qPCR analysis. Other colon tissues were kept at room temperature in 4% paraformaldehyde solution overnight.

### Histopathology analysis of colon

The samples were washed with BBS distilled water and transferred to 70% ethyl alcohol for long-term storage. Afterwards, the colon tissues were embedded in paraffin and sectioned at five μm thickness (Leica, RM2245). Hematoxylin and 0.5% eosin were used for histopathological staining (Leica, ST5010). Under a light microscope (Leica, DM2000), tissue sections were viewed at a magnifying power of ^*^100. Each group underwent four biological verifications.

### Reverse transcription-quantitative polymerase chain reaction (RT-qPCR)

The mRNA levels of ZO-1, Occludin-1, and Claudin-1 were measured by RT-qPCR. TRIzol reagent (Life, 15596-018) was used to extract RNA from snap-frozen colon tissues of each group. We performed RT-qPCR using the qTOWER 2.0 Real-Time PCR System (ABI, step one plus). The relative quantification of mRNA expression for ZO-1, Occludin-1, and Claudin-1 was analyzed via the 2^ΔΔ^Ct method following normalization with Gapdh. The specific primers were designed as follows: ZO-1, TGTCAGCCCTTCTGATGGTG (forward), and CTTCTTTGGCTGCAGGGCTA (reverse); Occludin-1, CATGGCGGCCTTTTGCTTCA (forward) and CCCAGGATTGCGCTGACTAT (reverse); Claudin-1, TCGGCTCTATCGTCAGCAC (forward) and GGACATCCACAGTCCCTCTG (reverse).

### Intestinal microbiota analysis

The 16S rDNA amplicon sequencing was executed by Novogene Corporation (Beijing, China). The total genome DNA was derived from colon content samples by CTAB. 1% agarose gels were used to measure DNA concentration and purity. The 16S rDNA V3-V4 regions were performed using the primers 341F (5′-CCTAYGGGRBGCASCAG-3′) and 806R (5′-GGACTACNNGGGTATCTAAT-3′). Sequencing libraries were prepared using NEBNext® Ultra™ IIDNA Library Prep Kit (Cat No. E7645). The Agilent Bioanalyzer 2100 system and Qubit@ 2.0 Fluorometer (Thermo Scientific) were used to assess the quality. Lastly, 250 bp paired-end reads were elicited by Illumina on a NovaSeq platform. FLASH (Version 1.2.11) was used for gaining Raw Tags (22) and Fastp (Version 0.20.0) was used for obtaining Clean Tags. By measuring the Clean Tags against the Silva database, the chimera sequences were identified using Vsearch (Version 2.15.0) and removed to winnow out the Effective Tags (23). Then, using the deblur module in the QIIME2 software (Version QIIME2-202006), initial ASVs (default: DADA2) were obtained, and then ASVs with an abundance of <5 were filtered out (24). As a result, the absolute abundance of ASVs was normalized by using the least abundant sample as the standard.

### Statistics

SPSS version 22.0 was used for statistical analysis. The values were presented as the mean ± *SD*. Multiple-group comparisons were assessed through one-way ANOVA and Tukey tests. *P* < 0.05 was considered statistically significant. Unless otherwise stated, the data were processed with GraphPad Prism 9.3.1. The 16S rDNA data were run on the Omicshare Platform (www.omicshare.com).

## Results

### Effects of abdominal massage on alleviating diarrhea

**Figure 2:**
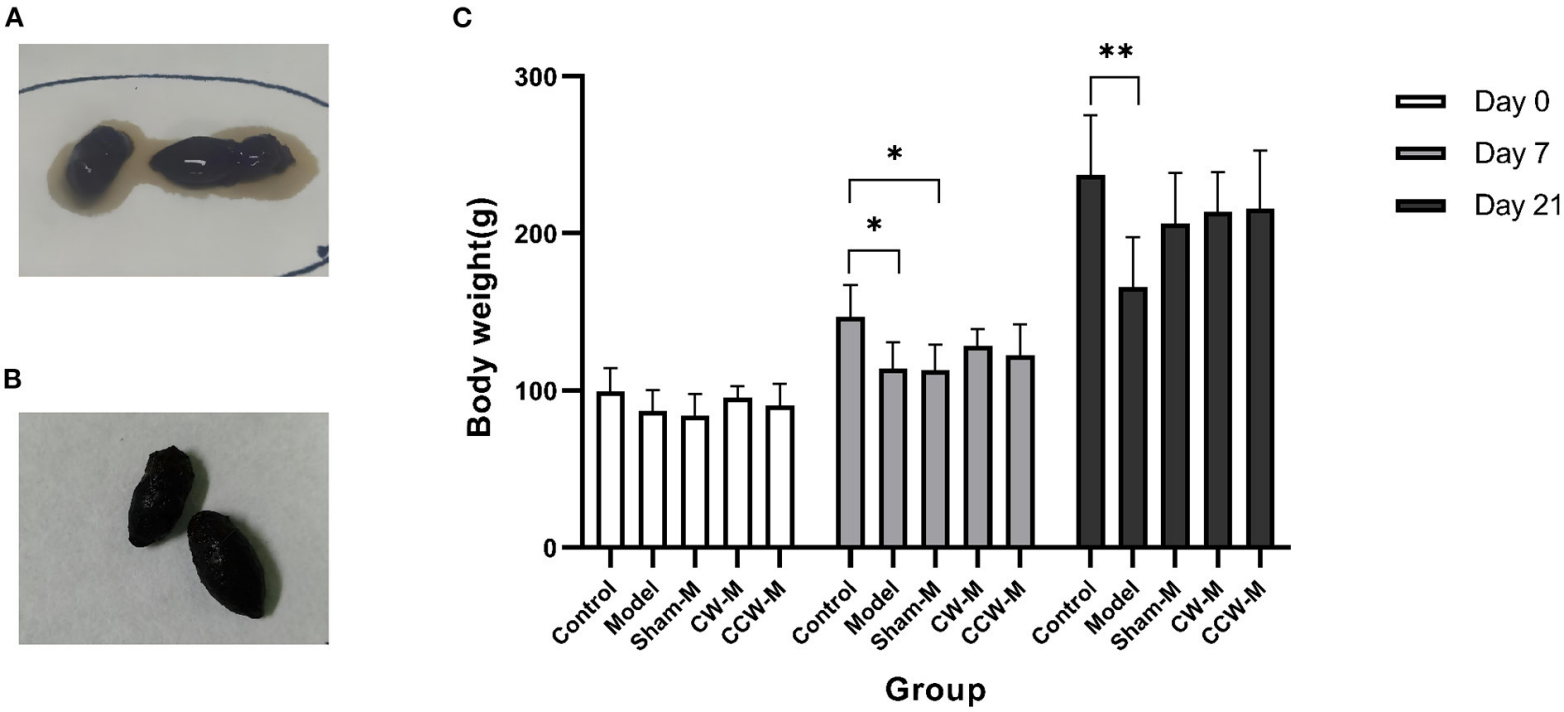
**(A)** State of stool before abdominal massage. **(B)** State of stool after abdominal massage. **(C)** Body weight. Error bars are expressed as means ± *SD* (*n* = 6). Statistical significance was determined by one-way ANOVA with Tukey tests for multiple-group comparisons. *P < 0.05; **P < 0.01.

The effects of abdominal massage on functional diarrhea were investigated by massaging immature rats for 2 weeks. Except for the control group, watery stools or diarrhea developed in all of the groups after the first day of Folium Sennae and Rhubarb ingestion (Figure 2A). After 2 weeks of abdominal massage, the stools of both CW and CCW groups of massage were normalized (Figure 2B).

We measured body weight each day. At the beginning of the experiment, all groups of rats had similar weights (*P* > 0.05). After 7 days of feeding with Folium Sennae and Rhubarb, we observed that the rats in the model had significantly less weight than the rats in the control group (*P* > 0.05). Compared to the control group, the growth rates of weight in other groups were slower. By the end of the massage (Day 21), no significant differences in weight were found between the sham massage, CW massage, and CCW massage groups. The mean weights in comparison were as follow: control group > CW-massage group > CCW-massage group > sham-massage group > model group (Figure 2C).

### Effects of abdominal massage on reversing intestinal barrier disruption

**Figure 3:**
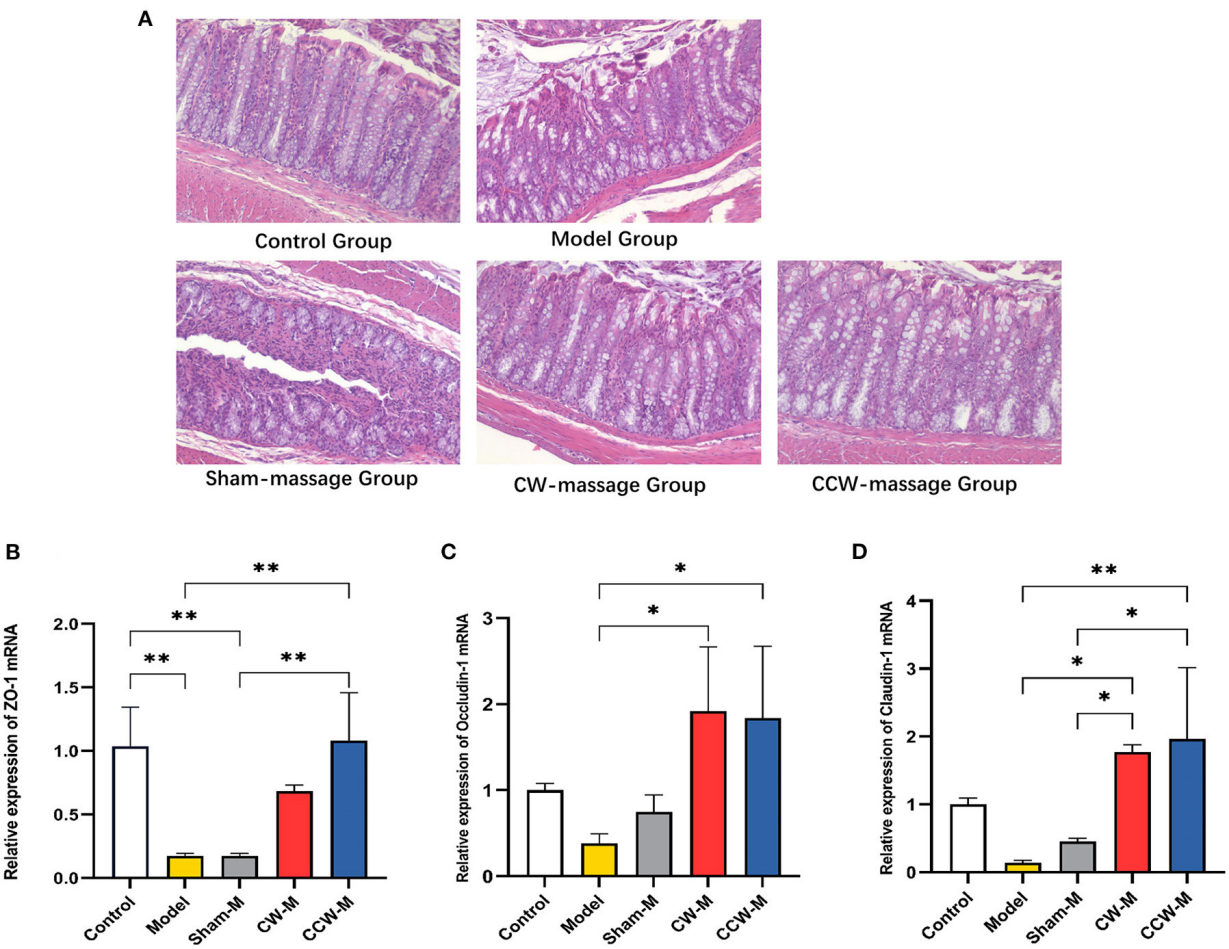
**(A)** H&E staining of colon tissue sample sections of rats. **(B–D)** Relative expression of ZO-1, Occludin-1 and Claudin-1 mRNA in colon was assessed using RT-qPCR. Error bars are expressed as means ± *SD* (*n* = 3). Statistical significance was determined by one-way ANOVA with Tukey tests for multiple-group comparisons, *P < 0.05; **P < 0.01.

As demonstrated by histologic analysis and RT-qPCR assay, abdominal massage could restore the damaged intestinal barriers in FD rats. The HE staining of the colon revealed that the epithelial cells in the model group were exfoliated, necrosed, and not arranged neatly as compared to the control group. Also, the glands have partial atrophy as well as mucosal edema. The situation of the sham-massage group was similar to that of the model group. Conversely, two massage groups reversed the above situation partially. The villus structure was relatively intact, and mild submucosa edema was still observed (Figure 3A). Concomitantly, ZO-1, Occludin-1, and Claudin-1 mRNA expression levels were significantly increased in both massage groups (Figures 3B–D). These results indicate that abdominal massage has a significant effect on maintaining intestinal epithelium integrity.

### Effects of abdominal massage on eliminating intestinal microbiota dysbiosis caused by functional diarrhea

#### Species annotation and evaluation

A total of 110,677 raw reads were generated from each sample in this study. After discarding low-quality sequences, on average 77,611 clean tags were obtained. The average length of the tags was 419. These tags were then subjected to the following analysis and clustered into operational taxonomic units (ASVs).

#### Alpha analysis

Alpha diversity indexes were calculated to assess the richness, diversity, and coverage of the sample microbial community in response to abdominal massage. The Species Accumulation Boxplot has become steady, which indicated that enough sequencing data has been collected and was acceptable (Supplementary Figure 1A). All samples reflected 100% community coverage in their coverage indexes. Clearly, these results were reasonable and reflected the actual microbiological situation. However, richness and diversity did not differ significantly between the groups (*P* > 0.05, Supplementary Figures 1B–D).

#### Beta analysis

**Figure 4:**
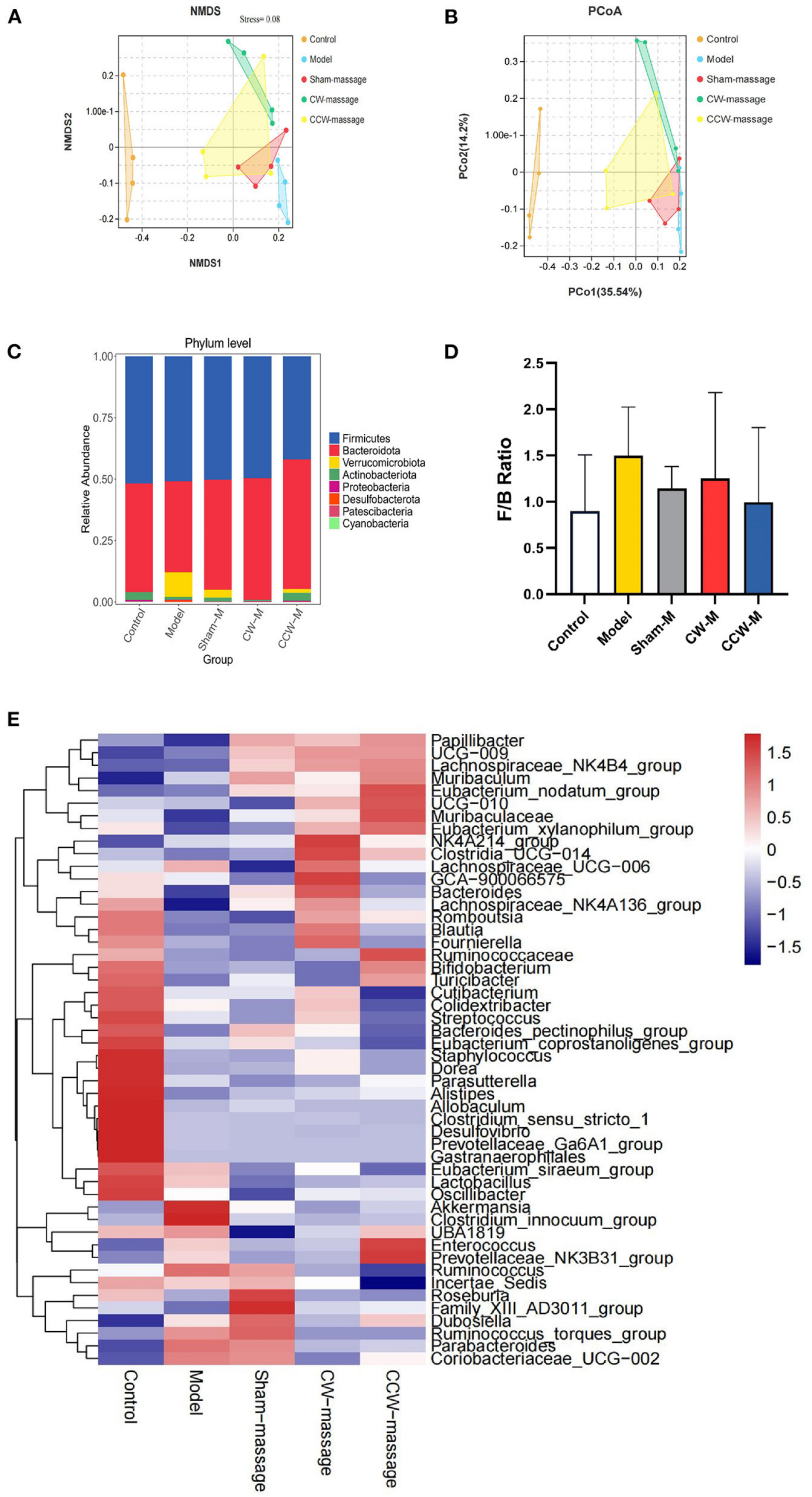
**(A)** NMDS plot of Bray-Curtis dissimilarity, Stress = 0.08. **(B)** PCoA plot of Bray-Curtis dissimilarity. **(C)** Relative abundance of ASVs at phylum level. **(D)** F/B Ratio in each group. Error bars are expressed as means ± SD (*n* = 4). Statistical significance was determined by one-way ANOVA with Tukey tests for multiple-group comparisons. **(E)** Heatmap of relative abundances at the genus level [top 50].

Additionally, the PCoA and NMDS of Bray-Curtis dissimilarity were used to assess beta diversity within each group. PCoA and NMDS revealed that microbiota structure in the model group was remarkably different from that in the control group. In contrast, the CW-massage and CCW-massage groups were restored to some extent (Figures 4A,B). Based on the analyses of the ANOSIM data, the differences between the five groups in terms of community composition were more apparent than those within each group (ANOSIM R = 0.5421, *P* = 0.001). These results further indicated that abdominal massage significantly altered the structural diversity of the intestinal microbiota of FD rats.

#### Altered composition of the intestinal microbiota

To investigate the impact of abdominal massage on the intestinal microbiota in FD rats, the phylum and genus abundance were examined. At the phylum level, *Firmicutes, Bacteroidota, Verrucomicrobiota, Actinobacteriota, Proteobacteria*, and *Desulfobacterota* were the dominant microbiota constituents (Figure 4C). In contrast to the control group, the model group exhibited an increment of *Firmicutes/Bacteroidetes* (F/B) ratio (*P* > 0.05, Figure 4D). The prevailing microbiota at the genus level were *Muribaculaceae, Dubosiella, Lactobacillus, Lachnospiraceae_NK4A136_group, Romboutsia, Bacteroides, Akkermansia, Ruminococcus*, and *Bifidobacterium* (Figure 4E).

**Figure 5:**
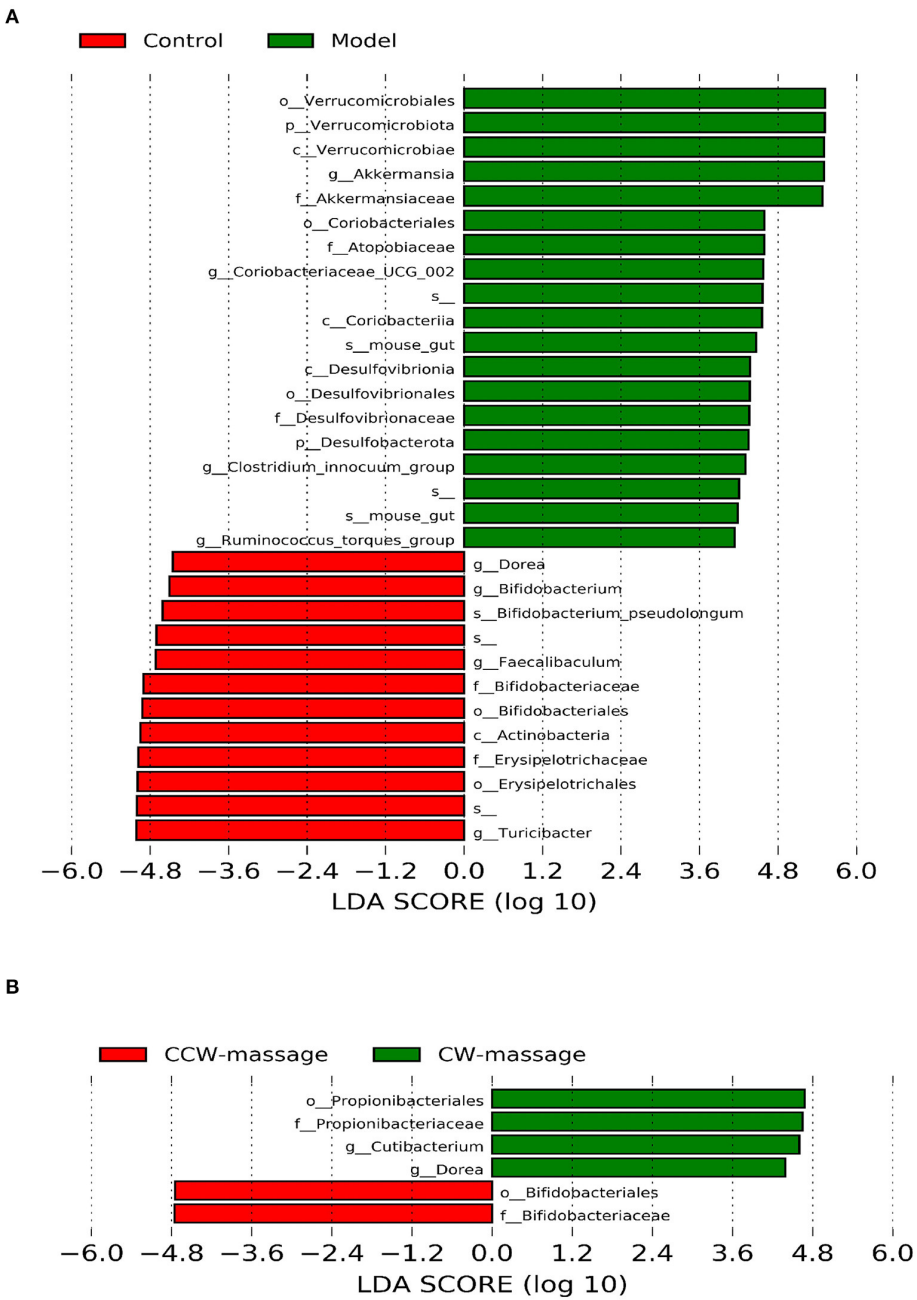
LDA effect size identifies differential abundance of bacteria between the control group and model group **(A)**, or between the sham-massage, CW-massage, and CCW-massage group **(B)**, LDA > 2.0, *P* < 0.05.

Specifically, LEfSe (LDA Effect Size) was used to analyze the differential enrichment of bacterial taxa between the control and model groups or between the sham-massage, CW-massage, and CCW-massage groups. Using a logarithmic cutoff score of 2.0, the following species showed statistically significant differences. In the control group, several genera were overrepresented, such as *Bifidobacterium, Faecalibaculum, Turicibacter*, and *Dorea*. While *Verrucomicrobiota, Akkermansia, Coriobacteriaceae-UGG-002, Desulfobacterota, Clostridium-innocuum-group*, and *Ruminococcus_torques_group* were gathered in the model group (Figure 5). Moreover, *Bifidobacteriaceae* were enriched in the CW-massage group, while *Cutibacterium, Propionibacteriaceae*, and *Dorea* were raised in the CCW-massage group.

**Figure 6:**
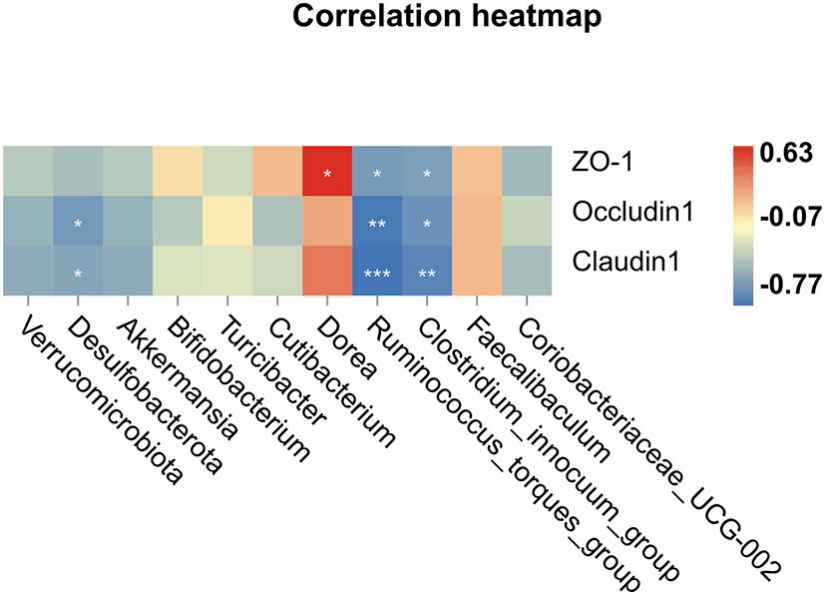
Spearman correlation analysis, *P < 0.05, **P < 0.01, ***P < 0.001.

Spearman correlations were performed to further determine the relationship between tight junction proteins in the colon and intestinal microbiota, which have meaningful correlations at the phylum and genus levels. A positive interaction was found between *Dorea* and ZO-1, while the two bacterial strains (*Ruminococcus_torques_group* and *Clostridium_innocuum_group*) were to be negatively interacted with ZO-1, Occludin-1, and Claudin-1 (Figure 6). We concluded that these bacteria were likely to play a significant role in the development of FD and its prevention.

#### COG annotation and analysis

**Figure 7:**
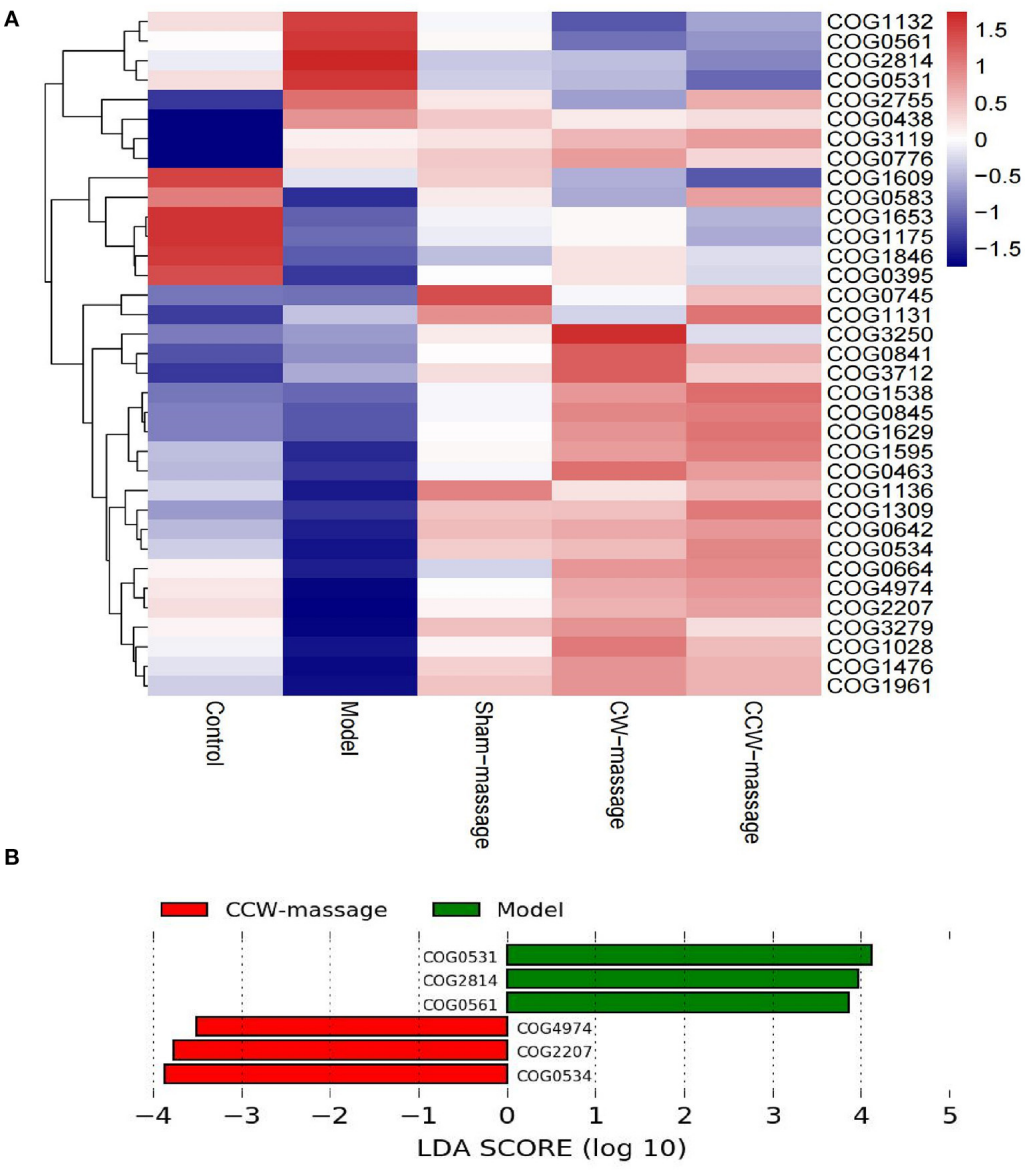
COG Annotation and Analysis. **(A)** Heatmap of the relative abundances of COG categories between five groups [top 35 functions]. **(B)** LDA integrated with LEfSe comparison of relative abundances of COG categories between five groups (LDA > 2.0, *P* < 0.05).

We aimed to predict the functional properties of the intestinal microbiota using the PICRUSt software and COG family information to further elucidate the possible role of the intestinal microbiota after FD and abdominal massage (Supplementary File 2). The top 35 functions for each group were chosen to create a heatmap. Also, the heatmaps were clustered according to functional levels. As shown in Figure 6, in the model group, five functional COG categories were decreased, including Transcription[K]; Cell wall/membrane/envelope biogenesis[M]; Signal transduction mechanisms[T]; Replication, recombination, and repair[L] and Defense mechanisms[V]. Then, significant differences in functional COG categories were identified using the LEfSe (Figure 7). We found six significantly different functional COGs between the model and CCW-massage group. The Model group exhibited a high enrichment of three functional COG categories, including Amino acid transport and metabolism[E], Carbohydrate transport and metabolism[G], and Coenzyme transport and metabolism, for General function prediction only[HR]. In contrast, the CCW group was enriched in Transcription[K], Replication, recombination and repair[L], and Defense mechanisms[V].

## Discussion

It is well-known that the balance of intestinal microbes plays a crucial role in maintaining the structural integrity of the gastrointestinal mucosal barrier, immunomodulation, and protection against pathogens (25). According to numerous studies, diarrhea may contribute to microbial dysbiosis within the intestines (26). Dysbiosis is comprised of the fall of key taxa, the reduction in taxonomic diversity, metabolic changes, and the increase in pathogenic germs (27). As suggested by previous studies, FD is related to the change of intestinal microbiota (9). Through Beta analysis, we found that community structures were significantly different between the model and control groups. Histological analysis and RT-qPCR assays have demonstrated that FD disrupts the integrity of the intestinal barrier. These observations suggest that FD is probably brought about by intestinal microbiota dysbiosis and disruption of the intestinal barrier. Moreover, In contrast to Irritable Bowel Syndrome or Antibiotic-Associated Diarrhea (28, 29), the FD model rats did not exhibit an overgrowth of colitis-associated bacteria, such as *Enterobacter, Salmonella*, and *Escherichia coli*. This finding suggests that FD is not associated with bacteria that cause intestinal infections. It should be noted that according to recent studies, *Akkermansia* may have a series of protective effects, including the stimulation of mucosal microbial networks and improvements in intestinal barrier function (30). In this study, *Akkermansia* was enriched in the model group. Rhubarb could be the reason for this phenomena, since Rhubarb has been found to contain ingredients that could increase the content of *Akkermansia* (31, 32). Therefore, it is not ruled out that Rhubarb could have been interfering with the experimental results.

Abdominal massage has gained more attention in clinics as a complementary therapy. In previous studies, continuous abdominal massage was shown to be able to improve intestinal transit and alleviate visceral hypersensitivity in diarrhea rats (33). In our study, we also discovered that the form of feces and the condition of colonic tissue significantly recovered after abdominal massage.

At the microbiota level, we found that intestinal microbiota was notably changed after abdominal massage. In the Beta analysis (Figures 4A,B), the two massage groups were able to close the distance with the control group in the community structure. These results corroborate that massage can modulate intestinal microbiota to an extent. LEfSe analysis showed that *Dorea, Bifidobacterium* were enriched to a greater extent, and *Clostridium_innocuum_group, Coriobacteriia, Ruminococcus_torques_group* were decreased after abdominal massage. To further explore, we investigated the relationship between tight junction protein indexes of the colon and the intestinal microbiota. Epithelial cells were connected through tight junctions to form the mucosal barrier in the intestine. And the integrity of the barrier was heavily dependent on the preservation of tight junction proteins (including ZO-1, Occludin-1, and Claudin-1) (34). In addition, the intestinal microbiota can directly affect epithelial permeability via tight junction protein under normal and pathological conditions (35). It was demonstrated by RT-qPCR that both massage directions could normalize the mRNA expression of ZO-1, Occludin-1, and Claudin-1. Furthermore, Spearman correlation analysis indicated that *Ruminococcus_torques_group* and *Clostridium_innocuum_group* were in a strikingly negative relation to tight junction proteins. Observations in this study agreed with those in other studies, which have found a negative correlation between *Ruminococcus_torques_group* abundance and tight junction protein abundance (36, 37). In part, this could be explained by *Ruminococcus_torques_group* and *Clostridium_innocuum_group* participating in the disruption of the intestinal barrier. The toxic metabolite of *Clostridium* has been previously reported to strengthen oxidative damage in intestinal epithelial cells and compromised the epithelial layer lining the gut (38). Also, the proliferation of *Ruminococcus_torques_group* can debase the mucin from the mucous layer of epithelial cells on the surface of the intestine, which may be one of the causes of the breakdown of the intestinal barrier (39). These results demonstrate that abdominal massage can partly mitigate FD by reshaping the composition of the intestinal microbiota, decreasing the number of bacteria that damage intestinal mucosal barriers, and restoring the integrity of the intestinal barrier. We speculate that the following mechanism may contribute to its potential effect in altering the intestinal microbiota structure. To begin with, the gastro-intestinal (GI) tract contains numerous mechanosensitive circuits, which are essential to normal gut functioning (40). Secondly, abdominal massage is soft and gentle contact with the skin. The gentle action of this treatment can directly affect the abdomen muscles, stimulate the GI mechanosensitive circuits, adjust the function of the gastrointestinal autonomic nerves, and promote regular peristalsis of the gastrointestinal smooth muscles (41). Furthermore, this beneficial change can alter the composition of vital micronutrients in the gut and ultimately change the microbial composition and structure (42). The mechanism described above may partly explain the positive effects of abdominal massage. Further research is needed to examine the mechanism of action of abdominal massage on intestinal microbiota in the future.

The final step was to use PICRUSt2 to predict functional analysis of the COG database in each group. According to the literature, disturbances in intestinal microbiota function are associated primarily with perturbations in metabolic pathways (43). Our study confirmed this finding. Also, according to COG LEfSe analysis, we can infer that CCW-massage could balance three functions of the rats' intestinal microbiota, including Transcription[K]; Replication, recombination, and repair[L], and Defense mechanisms [V].

This study has certain limitations. It only revealed that abdominal massage improved FD by modulating intestinal microbiota and increasing intestinal tight junction expression. However, it's not clear how abdominal massage affected the microbiota. Therefore, more researches are needed to identify the underlying mechanisms between intestinal microbiota and abdominal massage.

In conclusion, our research demonstrates that FD is not associated with pathogen infection. A dysbiosis of the intestinal microbiota and disruption of the intestinal barrier are most likely to cause FD. Moreover, this study supports that abdominal massage can alleviate FD by altering the intestinal microbiota structure and reducing the number of bacteria detrimental to the intestinal mucosal barrier. Furthermore, the intestinal microbiota and tight junction proteins did not significantly change following treatment with two massage directions. This study confirmed that abdominal massage is an effective complementary therapy for children with FD and provided references for the clinical therapy of FD. Future studies should examine the mechanism of motion of abdominal massage on FD.

## Data availability statement

The original contributions presented in the study are included in the article/supplementary material, further inquiries can be directed to the corresponding authors.

## Ethics statement

The animal study was reviewed and approved by the Animal Experimentation Ethics Committee of Guangzhou University of Chinese medicine.

## Author contributions

YH and YS contributed to the concept and design of the study. YH, QM, and XL performed the main experiments. JXH and QXM performed a portion of the statistical analysis. YH wrote the first draft of the manuscript. HL, JYH, and YS have revised this manuscript. All authors have approved the final version before submission.

## Conflict of interest

The authors declare that the research was conducted in the absence of any commercial or financial relationships that could be construed as a potential conflict of interest.

## Publisher's note

All claims expressed in this article are solely those of the authors and do not necessarily represent those of their affiliated organizations, or those of the publisher, the editors and the reviewers. Any product that may be evaluated in this article, or claim that may be made by its manufacturer, is not guaranteed or endorsed by the publisher.
