# Supplementary figures and images for: Abdominal massage alleviates functional diarrhea in immature rats via modulation of intestinal microbiota and tight junction protein
Source: Front Pediatr. 2022 Jul 22;10:922799. doi: 10.3389/fped.2022.922799 (PMC9354804; doi:10.3389/fped.2022.922799)

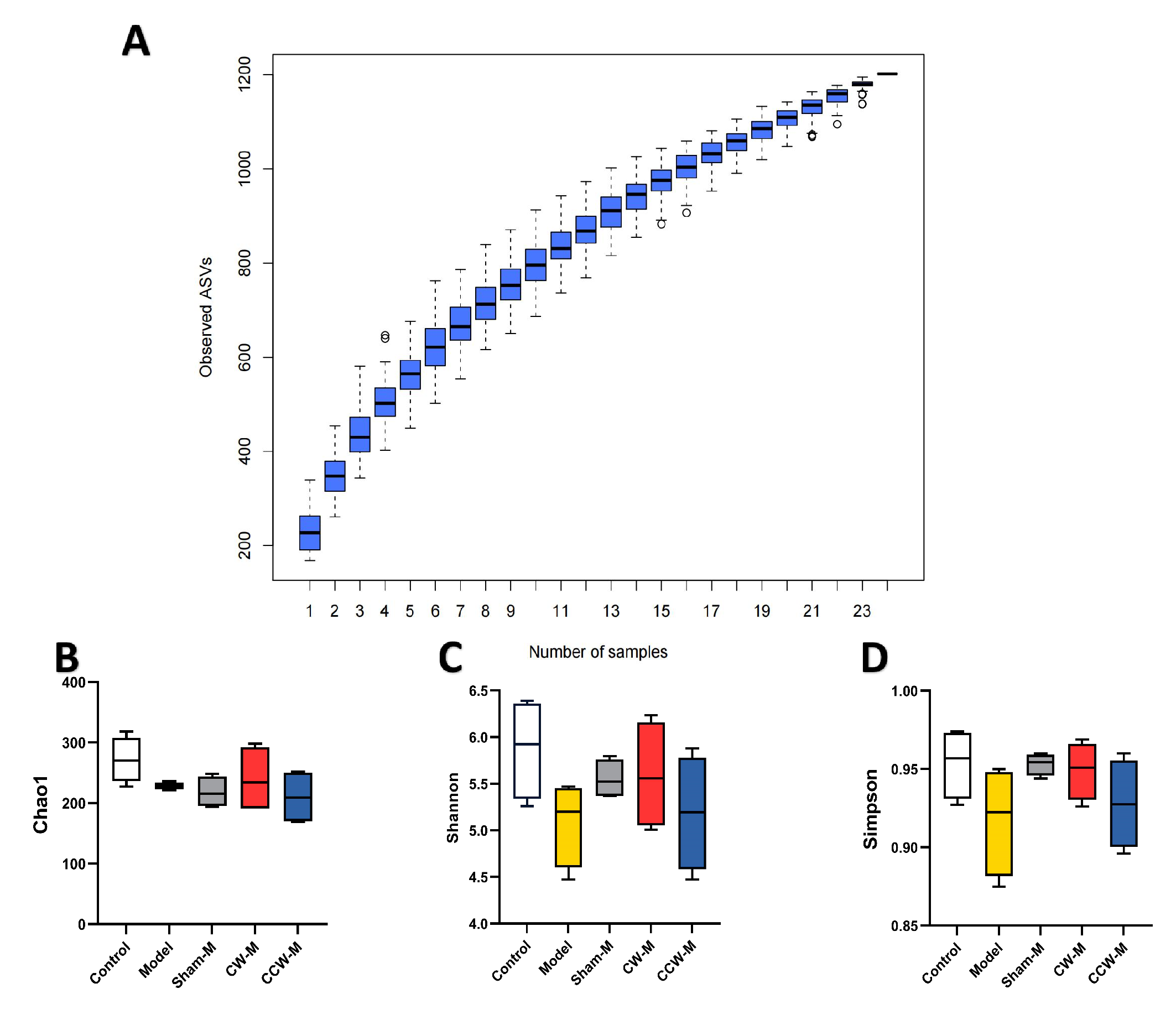

Supplement: Supplementary file 2 [file Image_1.TIFF]
